# Chemoenzymatic synthesis and identification of medium- and long-chain triacylglycerol congeners

**DOI:** 10.1007/s10068-025-02006-7

**Published:** 2025-10-24

**Authors:** Jaehyeon Park, Juno Lee, Jihoon Kim, Pahn-Shick Chang

**Affiliations:** 1 Department of Agricultural Biotechnology, Seoul National University, Seoul, 08826 Republic of Korea; 2 Research Institute of Agriculture and Life Sciences, Seoul National University, Seoul, 08826 Republic of Korea; 3 Center for Agricultural Microorganism and Enzyme, Seoul National University, Seoul, 08826 Republic of Korea; 4 Center for Food and Bioconvergence, Seoul National University, Seoul, 08826 Republic of Korea

**Keywords:** Structured lipids, Medium- and long-chain triacylglycerol, Regioisomer, Steglich esterification, Immobilized lipase, HPLC–ESI–MS, NMR spectroscopy

## Abstract

Medium- and long-chain triacylglycerols (MLCTs) offer rapid energy release and essential nutritional benefits, making them valuable in functional foods. In this study, MLCTs containing caprylic acid (C8:0; C) and linoleic acid (C18:2; L) were synthesized via a chemoenzymatic strategy combining lipase-catalyzed reactions and Steglich esterification. Four distinct MLCT congeners—CCL, CLC, LLC, and LCL—were obtained with yields of 52–67% (asymmetrical) and 71–84% (symmetrical), and purified using preparative HPLC (> 98%). The resulting compounds were structurally characterized by HPLC–ESI–MS and ^13^C NMR spectroscopy. HPLC–ESI–MS confirmed the expected mass-to-charge values for the [M + NH₄]⁺ adducts: 624.6 for CCL/CLC and 760.6 for LLC/LCL. ^13^C NMR analysis verified molecular symmetry; asymmetric TAGs showed three carbonyl signals (e.g., 173.30, 173.26, 172.88 ppm for CCL), while symmetric forms showed two signals. These results confirm the successful synthesis and structural integrity of MLCTs with defined fatty acid positioning for functional lipid applications.

## Introduction

Structured lipids (SLs) are a class of modified triacylglycerols (TAGs) designed to possess improved nutritional, functional, or metabolic properties by controlling the type (e.g., chain length and unsaturation degree) and positional distribution of fatty acids on the glycerol backbone (Iwasaki and Yamane, 2000). Through precise structural modification, SLs can be tailored to fulfill specific physiological roles, such as enhanced absorption, improved energy utilization, and/or delivery of essential fatty acids (Guo et al., 2020). Among various types of SLs, medium- and long-chain TAGs (MLCTs) have attracted growing attention due to their dual functionality derived from their fatty acid composition (Lee et al., 2022).

MLCTs are composed of both medium-chain fatty acids (MCFAs, typically C6–C12) and long-chain fatty acids (LCFAs, ≥ C14). MCFAs are more rapidly hydrolyzed by human pancreatic lipase and absorbed via the portal vein, providing quick energy without significant fat accumulation (Nimbkar et al., 2022). On the other hand, LCFAs play crucial roles in membrane structure, signaling pathways, and the delivery of essential fatty acids (Khan et al., 2023). By combining these two classes of fatty acids within a single TAG molecule, MLCTs offer metabolic advantages that are not observed with conventional TAGs (Wang et al., 2022). Consequently, MLCTs are being widely explored for applications in clinical and personalized nutrition, sports supplements, infant formula, and functional food formulations with tailor-made purposes (Kasai et al., 2003; Osborn and Akoh, 2002; Wang et al., 2025).

However, the biological efficacy of MLCTs is strongly influenced by the positional distribution (*sn*-1, *sn*-2, and *sn*-3 positions) of fatty acids on the glycerol backbone (Jiang et al., 2022). It is well established that human pancreatic lipases exhibit strong positional specificity, hydrolyzing ester bonds at both *sn*-1 and *sn*-3 positions while largely preserving the *sn*-2 ester bond (Lim et al., 2022). Therefore, the incorporation of bioactive or digestively favorable fatty acids at specific positions (e.g., LCFAs at *sn*-2 position or MCFAs at *sn*-1,3 positions) is essential to direct their digestion, absorption, and metabolic utilization (Utama et al., 2019). This highlights the need for synthetic strategies that can introduce fatty acids into specific positions of the TAG structure.

Enzymatic synthesis using immobilized lipases with regioselectivity (e.g., Lipozyme RM IM, Lipozyme TL IM, and Lipozyme 435) has been widely applied for the preparation of SLs, offering high selectivity under mild reaction conditions and avoiding the formation of undesirable by-products (Kim and Akoh, 2015). However, enzymatic methods are sometimes limited by low yields, side reactions (e.g., hydrolysis), or extended reaction times (Remonatto et al., 2022). Conversely, chemical esterification techniques, such as Steglich esterification, enable broader substrate use and faster reaction rates, but typically lack positional control (Munawar et al., 2024). To overcome these individual limitations, chemoenzymatic synthesis has emerged as an attractive strategy, combining the regioselectivity of lipase-catalyzed reactions with the flexibility and efficiency of chemical coupling methods (Haraldsson et al., 2000). This integrated approach holds the potential for selective synthesis of MLCT congeners, achieving greater efficiency and environmental sustainability than previous methods alone.

In the present study, we applied this chemoenzymatic approach to synthesize four structurally distinct MLCT congeners containing caprylic acid (C8:0; denoted as C) and linoleic acid (C18:2 *cis*-9, *cis*-12; denoted as L): CCL, CLC, LLC, and LCL. The selection of C and L was based on their functional significance to MLCT research, with C exhibiting oxidative stress-reducing effects and L promoting skin repair (Cansız et al., 2021; Simard et al., 2022). These two fatty acids were incorporated into four MLCT congeners–two asymmetrical (CCL, LLC) and two symmetrical (CLC, LCL)–to investigate the differences in synthetic methods and yields among the MLCT types. Asymmetric MLCTs (CCL, LLC) and symmetric MLCTs (CLC, LCL) were synthesized using monoacylglycerol (MAG) via lipase-catalyzed esterification or ethanolysis based on regioselective reaction, followed by subsequent Steglich esterification to complete the targeted congeners. Finally, the regioisomerism of MLCT congeners was confirmed by the number of carbonyl resonances corresponding to *sn*-1, *sn*-2, and *sn*-3 positions, enabling clear differentiation between symmetric and asymmetric TAGs. This work provides a systematic methodology for the controlled synthesis and structural verification of MLCTs, offering a promising platform for the development of structured lipids with tailored physiological functionality.

## Materials and methods

### Materials

Tricapryloylglycerol (TCG, ≥ 95%), trilinoleoylglycerol (TLG, ≥ 97%), linoleic acid (≥ 98%), and DL-1,2-isopropylideneglycerol (≥ 97%) were purchased from Sigma-Aldrich (St. Louis, MO, USA), and caprylic acid (≥ 98%) from Junsei Chemical (Tokyo, Japan). N, N-Dicyclohexylcarbodiimide (DCC, ≥ 98%), 4-dimethylaminopyridine (DMAP, ≥ 99%), dichloromethane (DCM, HPLC grade), and hydrochloric acid (HCl) were purchased from Daejung Chemical & Metals Co., Ltd (Gyeonggi-do, Republic of Korea). Lipozyme 435 (lipase B from *Candida antarctica* immobilized onto macroporous acrylic resin) with a catalytic activity of 10,000 propyl laurate units/g was kindly donated by Novozymes (Bagsvaerd, Denmark). Ethanol (EtOH, HPLC grade), chloroform (HPLC grade), acetonitrile (HPLC grade), *n*-hexane (HPLC grade), 2-propanol (HPLC grade), trifluoroacetic acid (TFA, guaranteed reagent grade), and sodium sulfate (≥ 98%) were purchased from Samchun Pure Chemicals (Gyeonggi-do, Republic of Korea). Methanol (HPLC grade) was purchased from J.T. Baker (Phillipsburg, NJ, USA), and sodium hydroxide (NaOH) from Duksan Pure Chemical Co., Ltd (Gyeonggi-do, Republic of Korea). Sodium bicarbonate (≥ 99%) was purchased from Kanto Chemical Co., Inc. (Tokyo, Japan). All other chemicals were of extra pure grade and used without further purification.

### Enzymatic synthesis of MAGs

#### Synthesis of 1-*rac*-MAGs

**Fig. 1:**
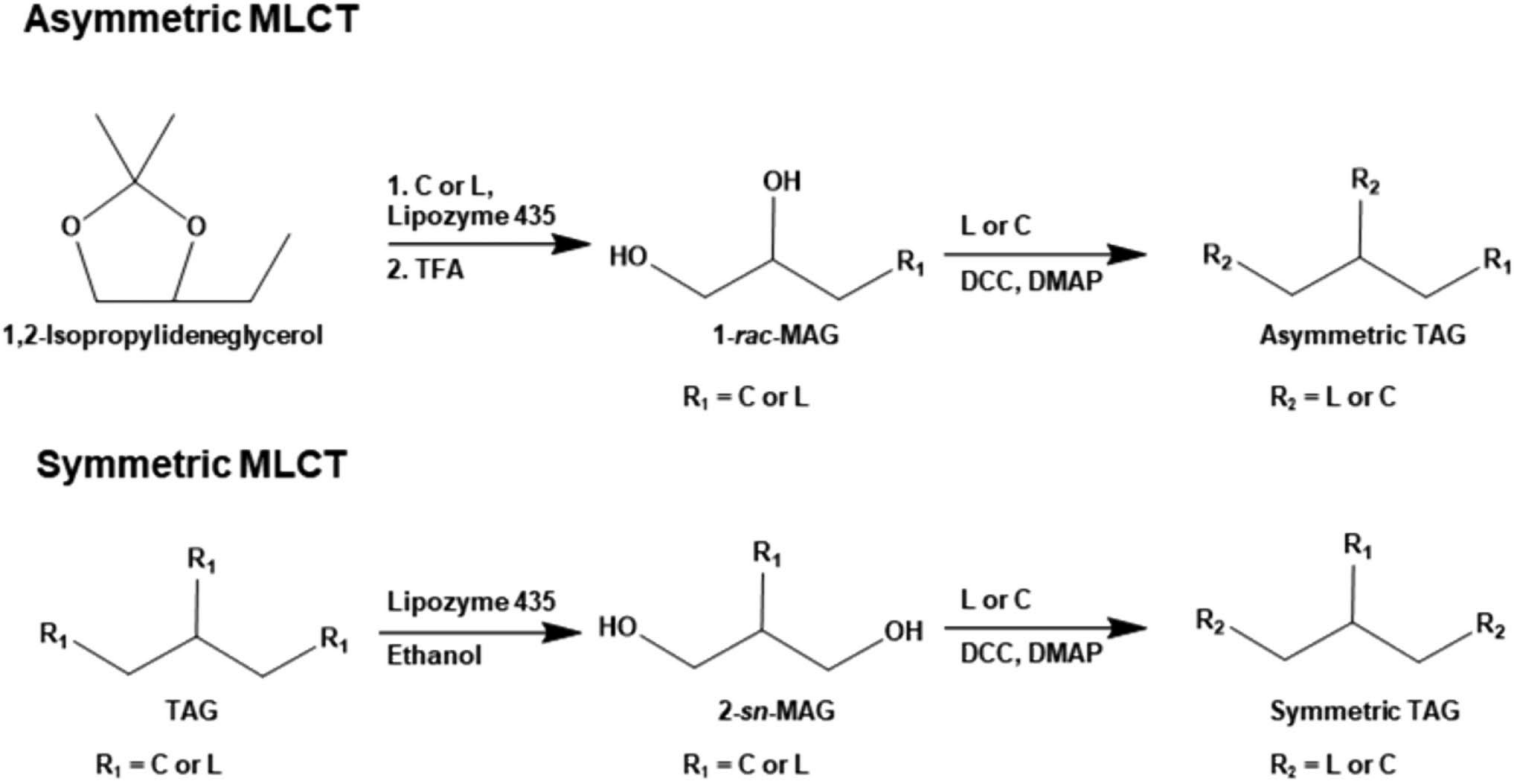
Scheme of chemoenzymatic synthesis of medium- and long-chain triacylglycerols (MLCTs). *C* caprylic acid, *L* linoleic acid, *TAG* triacylglycerol, *MAG* monoacylglycerol, *TFA* trifluoroacetic acid, *DCC* N, N-dicyclohexylcarbodiimide, *DMAP* 4-dimethylaminopyridine

1-*rac*-MAGs were synthesized with slight modifications from a previously reported method (Iwasaki et al., 2001; Kozlov et al., 2023) (Fig. 1). Caprylic acid or linoleic acid (0.3 mmol) was reacted with dl-1,2-isopropylideneglycerol (0.3 mmol) in the presence of Lipozyme 435 and water, which were added at 10% and 3% (w/w) of the total mass of the reaction mixture, respectively. The reaction was carried out at 40 °C and 300 rpm for 1 h. After the reaction, the enzyme was removed by filtration through a 0.45 μm PTFE syringe filter. Residual water was eliminated by treatment with molecular sieve 4 Å, and the resulting acylisopropylideneglycerol was collected. To deprotect the isopropylidene group, the sample was incubated in 90% trifluoroacetic acid at − 20 °C for 30 min, followed by neutralization with 40 mL of ice-cold 2 N NaOH. To extract the resulting MAGs, 50 mL of chloroform/methanol (4:1, v/v) was added, and the lower organic phase was collected.

#### Synthesis of 2-*sn*-MAGs

Based on a previous study (Irimescu et al., 2002; Palacios et al., 2022), 2-*sn*-MAGs were obtained through *sn*-1,3 specific ethanolysis (Fig. 1). TCG or TLG (0.4 mmol) was mixed with 30.8 mmol ethanol and stirred at 300 rpm for 10 min, followed by the addition of Lipozyme 435 (10%, w/w of the substrate). The ethanolysis reaction for TLG was performed for 4 h, based on the optimal reaction time reported in the previous study, while that for TCG was conducted for 2 h, as determined through preliminary time-course experiments (Irimescu et al., 2002). After the reaction, ethanol was evaporated under a stream of nitrogen gas, and the residue was redissolved in 4.0 mL of acetonitrile/water (95:5, v/v). The solution was washed three times with 4.0 mL of *n*-hexane to remove the formed ethyl esters. After solvent evaporation, the residue was dissolved in 4.0 mL of chloroform. To eliminate glycerol, 4.0 mL of water/ethanol (90:10, v/v) was added, and only the lower organic phase was collected.

#### Chemical synthesis of MLCTs

The MAGs obtained were converted into a structured lipid containing two different fatty acids via Steglich esterification at 25 °C for 16 h (Février et al., 2001) (Fig. 1). The molar ratio of MAG:free fatty acid:DCC:DMAP was set at 1.00:2.50:2.75:0.25. Although the stoichiometric requirement of free fatty acid is 2.00 equivalents (equiv) per 1.00 equiv of MAG, an excess amount (2.50 equiv) was used to ensure sufficient acyl donor availability and drive the esterification to completion. DCC, a coupling agent, was employed at 2.75 equiv to fully activate the carboxylic acid groups. DMAP was used as a nucleophilic catalyst in a catalytic amount (0.25 equiv), which is known to be sufficient for Steglich esterification reactions. Free fatty acid (C or L, 0.250 mmol) and DCC (0.275 mmol) were dissolved in 10 mL of ice-cold DCM and left to stand for 5 min. Subsequently, the synthesized MAG (0.100 mmol) and DMAP (0.025 mmol) were added, and the mixture was stirred at 25 °C for 16 h at 300 rpm. After the reaction, 10 mL of water was added and stirred for 30 min to hydrolyze the residual *O*-acylisourea intermediates into dicyclohexylurea. The aqueous layer was separated, and the organic phase was filtered through a 0.45 μm PTFE syringe filter to remove dicyclohexylurea. Remaining DMAP and unreacted free fatty acids were eliminated by five successive washes with 10 mL of 0.5 N HCl and 5% (w/v) sodium bicarbonate solution, respectively. Finally, residual moisture was removed by adding approximately 30 mg (3–6%, w/v) of sodium sulfate. The esterification products were analyzed using an HPLC (PU-4180; JASCO Co., Tokyo, Japan) equipped with an ultraviolet (UV) detector (UV-4075; JASCO Co.) and a silica-based Luna C18 column (5 µm, 4.6 × 150 mm; Phenomenex, Torrance, CA, USA). The samples were eluted under the following conditions: CCL/CLC with acetonitrile/2-propanol/acetic acid (90:10:0.1, v/v/v) at 1.0 mL/min, and LLC/LCL with acetonitrile/2-propanol/acetic acid (50:50:0.1, v/v/v) at 0.5 mL/min. The column temperature was maintained at 30℃. Finally, the reaction mixtures were analyzed by HPLC through injection of a 10 µL aliquot and monitored at 215 nm.

### Purification by preparative HPLC

The reaction mixture was purified through a preparative HPLC system (Ultimate 3000 Semi-Preparative System; Thermo Fisher Scientific) equipped with a Polaris 180 Å C18 column (5 µm, 21.2 × 250 mm; Agilent, Santa Clara, CA, USA). The mobile phase consisted of acetonitrile/2-propanol/acetic acid (90:10:0.1 and 50:50:0.1, v/v/v) for CCL/CLC and LLC /LCL, respectively, and was delivered at a flow rate of 5.0 mL/min. Fractions were collected based on UV absorbance at 215 nm and subsequently concentrated under a gentle stream of nitrogen gas.

### HPLC–ESI–MS

All compounds in the samples, filtered through a membrane filter (0.45 μm), were separated by an HPLC system (Ultimate 3000 RS; Thermo Fisher Scientific, Waltham, MA, USA) equipped with an ACQUITY UPLC BEH C18 column (1.7 μm, 2.1 × 100 mm; Waters Corporation, Milford, MA, USA) according to the previous method with slight modification (Park et al., 2023). The analytes were subjected to gradient elution with 0.1% (v/v) acetic acid and 10 mM ammonium formate in acetonitrile/water (6:4, v/v) (A) and 0.1% (v/v) acetic acid and 10 mM ammonium formate in acetonitrile/2-propanol (1:9, v/v) (B), as follows: 0.0–0.5 min, 5% B; 0.5–20.0 min, 5–100% B; 20.0–24.0 min, 100% B; 24.0–24.5 min, 100–5% B; 24.5–30.0 min, 5% B. The flow rate was maintained at 0.25 mL/min at all run times. Mass spectrometric analyses were performed using an LTQ XL linear ion-trap mass spectrometer (Thermo Scientific) with an ESI interface.

#### Nuclear magnetic resonance (NMR) spectroscopy

^13^C NMR spectroscopy was conducted using an AVANCE™ 600 MHz high-resolution NMR spectrometer (Bruker Corporation, Bremen, Germany) for structural analysis of the purified SLs, as described in a previous study with slight modifications (Kim et al., 2023). The samples were dissolved in deuterated chloroform (CDCl_3_) at a concentration of 20 mmol/L, with 2.15 mmol/L tetramethylsilane (TMS) used as an internal standard. Chemical shift values (*δ*) are expressed in parts per million (ppm) relative to the resonance of TMS. The NMR spectra were processed with TopSpin™ software (version 4.5.0; Bruker Corporation).

## Results and discussion

### Chemoenzymatic synthesis of MLCTs

To synthesize MLCTs, MAGs were first enzymatically prepared, followed by chemical esterification. As a result, 2-*sn*-MAGs can be readily obtained from TAG through ethanolysis using *sn*-1,3 regioselective lipases, yielding 2-*sn*-monocapryloylglycerol (MCG) and 2-*sn*-monolinoleoylglycerol (MLG) were obtained, with purities exceeding 95% after purification. In contrast, the preparation of 1-*rac*-MAGs via ethanolysis would require a lipase with *sn*-1,2 (or *sn*-2,3) stereospecificity, which is not feasible. Therefore, 1-*rac*-MAG was synthesized by first esterifying a fatty acid to isopropylidene glycerol, in which the *sn*-1,2 (or *sn*-2,3) positions are protected, followed by chemical deprotection. Using this method, 1-*rac*-MCG and 1-*rac*-MLG were obtained in 89% and 87% yields, respectively, both with purities greater than 95% following purification.

**Fig. 2:**
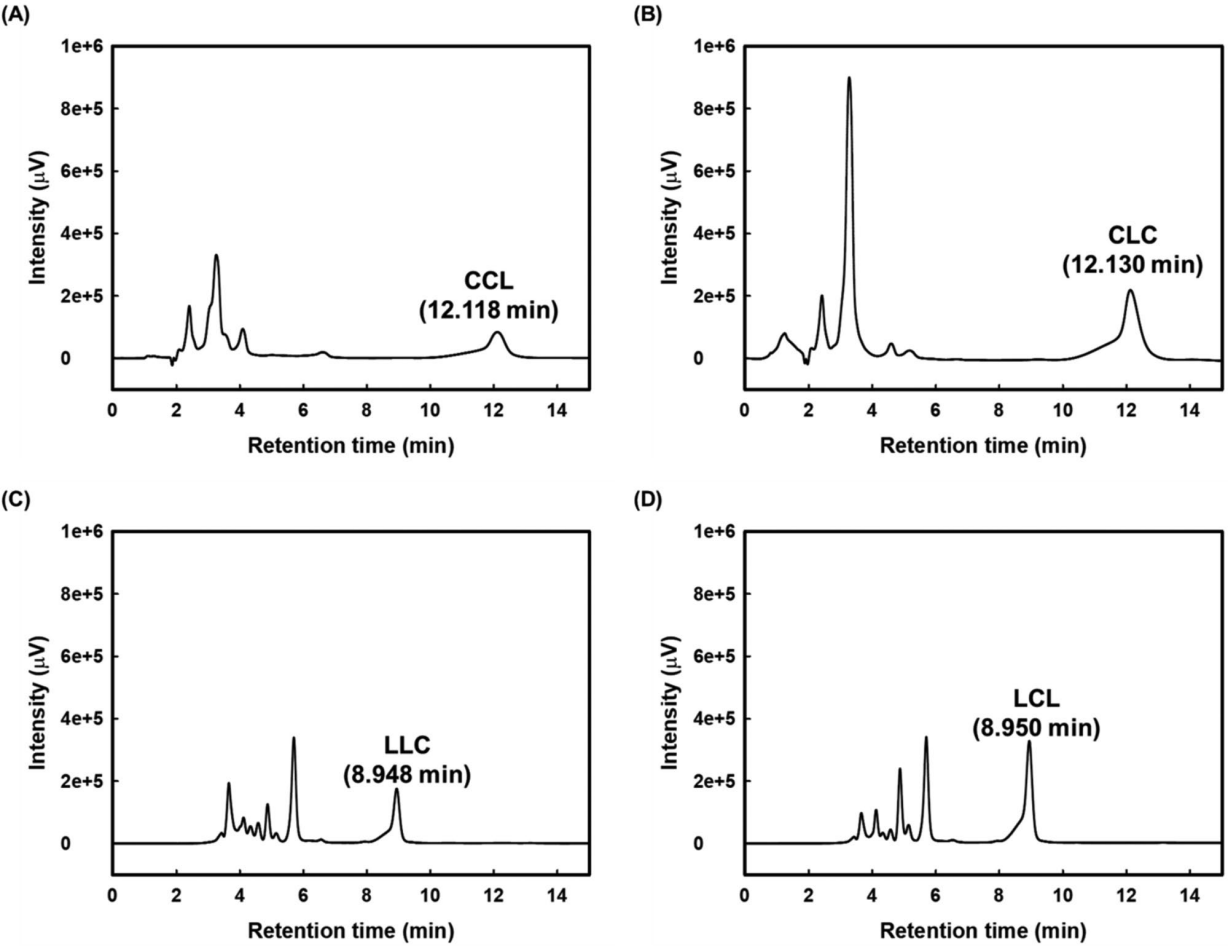
HPLC chromatograms of synthesized medium- and long-chain triacylglycerols. **A** 1,2-dicapryloyl-3-linoleoylglycerol (CCL), **B** 1,3-dicapryloyl-2-linoleoylglycerol (CLC), **C** 1,2-dilinoleoyl-3-capryloylglycerol (LLC), and **D** 1,3-dilinoleoyl-2-capryloylglycerol (LCL)

The asymmetric TAGs, CCL and LLC, were synthesized from 1-*rac*-MLG and 1-*rac*-MCG, respectively, while the symmetric TAGs, CLC and LCL, were derived from 2-*sn*-MLG and 2-*sn*-MCG, respectively. Figure 2 shows the chromatograms of each MLCT before purification. Under the mobile phase condition of acetonitrile/2-propanol/acetic acid (90:10:0.1, v/v/v) with a flow rate of 1.0 mL/min, CCL (67% yield) was detected at 12.118 min, while its positional isomer, CLC (84% yield), was eluted at a nearly identical retention time of 12.130 min. In contrast, LLC and LCL were not soluble in this solvent system and thus were analyzed using a mobile phase of acetonitrile/2-propanol/acetic acid (50:50:0.1, v/v/v) with a flow rate of 0.5 mL/min. Under these conditions, LLC (52% yield) was detected at 8.948 min, and its positional isomer, LCL (71% yield), was eluted at 8.950 min.

MLCTs composed of two linoleic acids and one caprylic acid (LLC, LCL) exhibited lower solubility in acetonitrile, a polar aprotic solvent, compared to that of two caprylic acids and one linoleic acid (CCL, CLC). This difference in solubility is likely attributed to the higher polarity of caprylic acid, which possesses a shorter carbon chain than linoleic acid (C8 < C18), resulting in lower hydrophobicity of the molecule and consequently higher solubility in acetonitrile (McKimmie et al., 2013). Moreover, symmetric MLCTs (CLC, LCL) exhibited higher synthetic yields than their asymmetric counterparts (CCL, LLC), which is consistent with a previous study (Février et al., 2001). This result may be due to the reduced steric hindrance and increased reaction uniformity associated with symmetric molecular structures. In the synthesis of symmetric TAGs, acylation of 2-*sn*-MAG at either the *sn*-1 or *sn*-3 position produces 1,2-*rac*-diacylglycerol (DAG), and the subsequent acylation at the remaining *sn*-1 or *sn*-3 position proceeds without significant steric hindrance, resulting in efficient TAG formation. In contrast, the synthesis of asymmetric TAGs from 1-*rac*-MAG can yield 1,2-*rac*-DAG or 1,3-*sn*-DAG. While acylation of 1,2-*rac*-DAG proceeds similarly to the symmetric TAG pathway, acylation of 1,3-*sn*-DAG at the *sn*-2 position may encounter steric hindrance from the fatty acids already occupying the *sn*-1 and *sn*-3 positions (Jordan et al., 2021). This structural constraint likely contributes to the generally lower yields for asymmetric TAGs compared to symmetric ones.

CCL and CLC, as well as LLC and LCL, are regioisomers that cannot be separated using a conventional C18 column under reverse-phase HPLC conditions, resulting in nearly identical retention times (Fig. 2). To overcome this limitation, we further structurally investigated the fatty acid composition of the synthesized TAGs using HPLC–ESI–MS analysis and distinguished the regioisomers using ^13^C NMR analysis.

### Purification of MLCTs

**Fig. 3:**
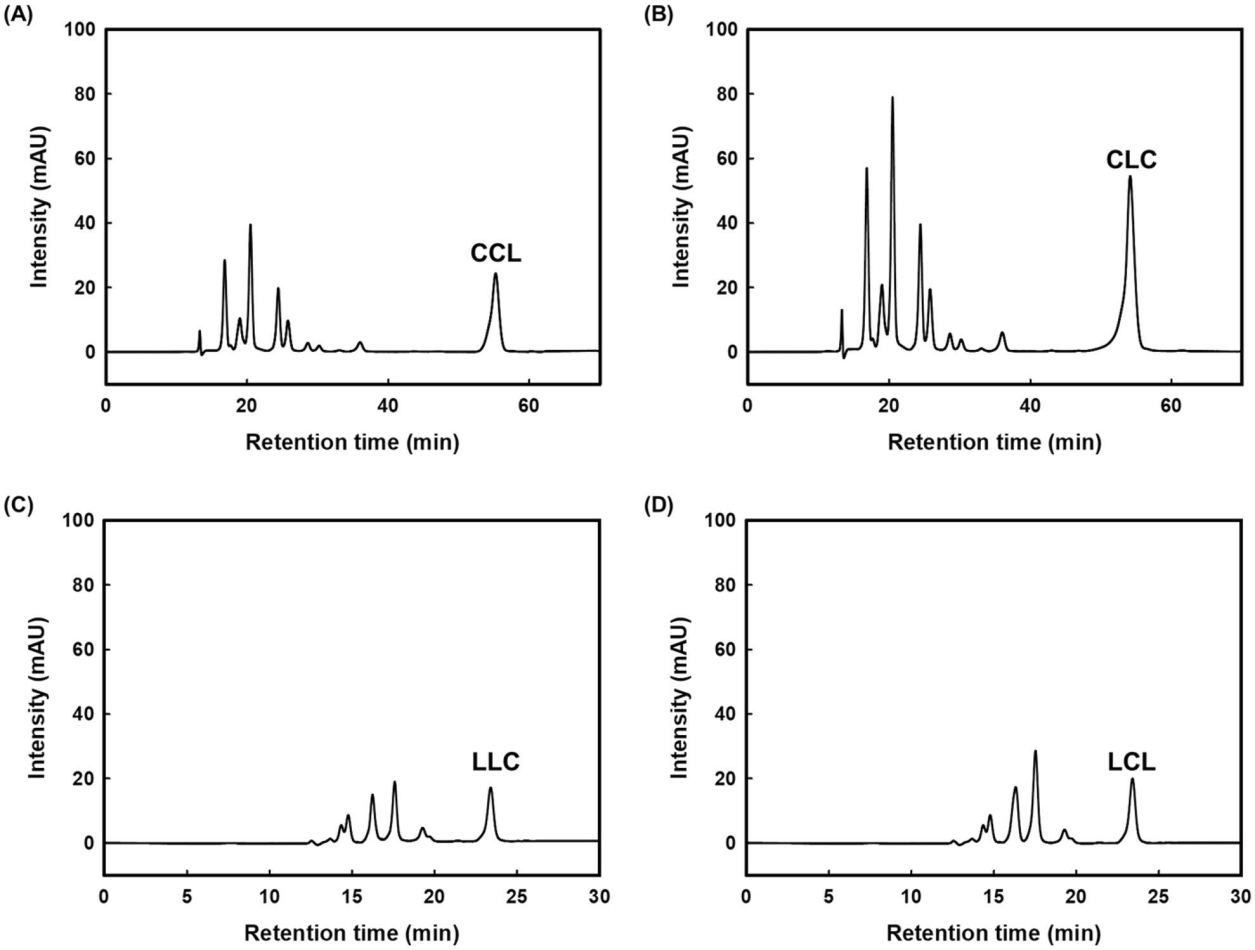
Preparative HPLC chromatograms of synthesized medium- and long-chain triacylglycerols. **A** 1,2-dicapryloyl-3-linoleoylglycerol (CCL), **B** 1,3-dicapryloyl-2-linoleoylglycerol (CLC), **C** 1,2-dilinoleoyl-3-capryloylglycerol (LLC), and **D** 1,3-dilinoleoyl-2-capryloylglycerol (LCL)

To obtain MLCTs with high purity, preparative HPLC was employed for purification. Based on analytical HPLC results, a mobile phase of acetonitrile/2-propanol/acetic acid (90:10:0.1 and 50:50:0.1, v/v/v for CCL/CLC and LLC/LCL, respectively) was employed at a flow rate of 5.0 mL/min. Figure 3 shows the preparative HPLC chromatograms of four MLCTs. CCL and CLC were detected at about 51 min, and the fractions collected between 50 and 53 min were pooled. Similarly, LLC and LCL were detected at about 24 min, and the corresponding fractions were collected from 21 to 25 min. During the collection periods, no peak overlap was observed between the target compounds and impurities, indicating that the desired MLCT congeners were successfully isolated with high purity (> 98%). After the complete removal of the solvent from the purified samples, all four MLCT products were found to be in the liquid state at 25 °C. This is likely due to the fact that both TCG and TLG, the parent compounds, are also liquids under the same conditions.

### Structural analysis of MLCTs

#### HPLC–ESI–MS analysis

**Fig. 4:**
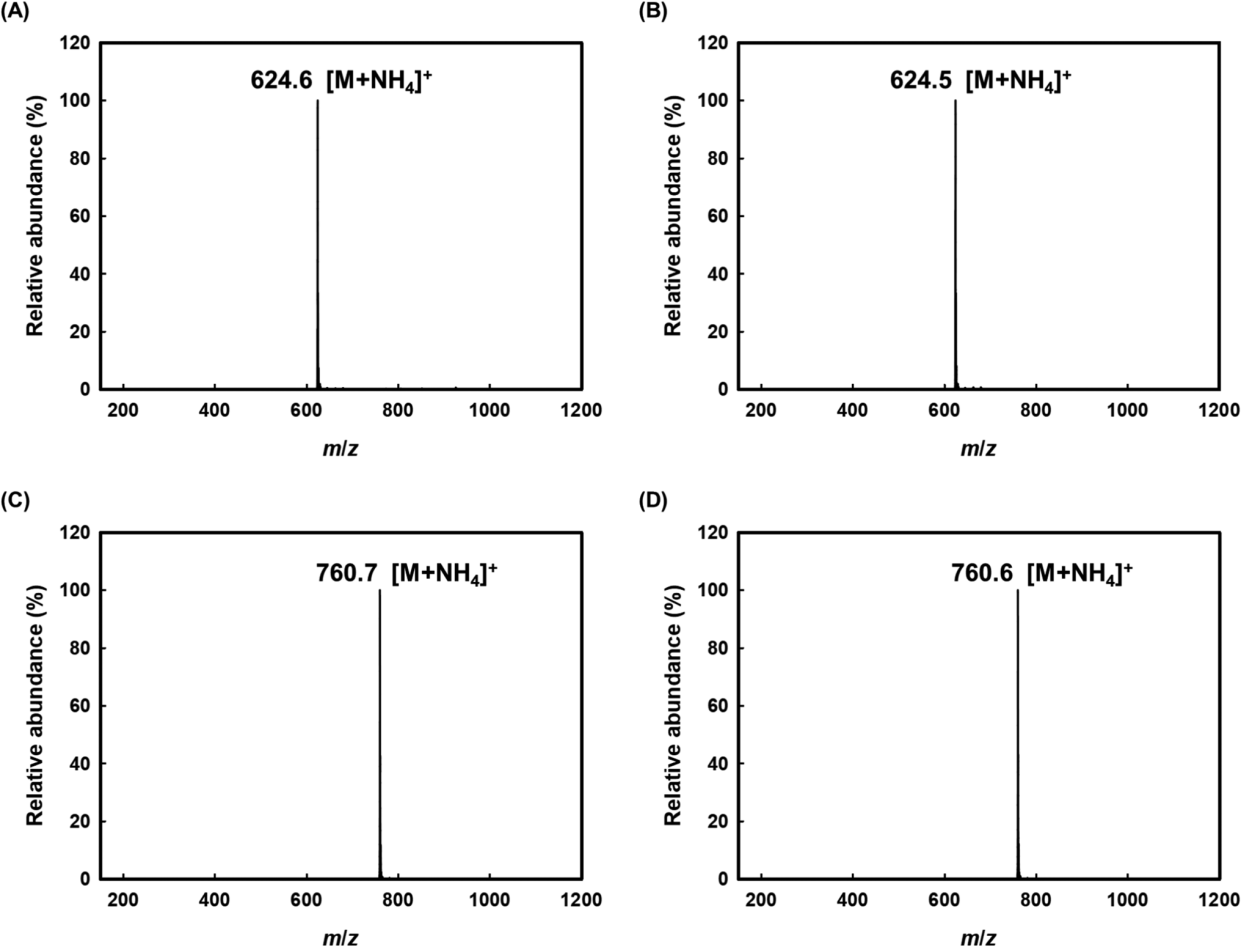
HPLC–ESI–MS spectra showing [M + NH_4_]^+^ adduct ions of medium- and long-chain triacylglycerols. **A** 1,2-dicapryloyl-3-linoleoylglycerol (CCL), **B** 1,3-dicapryloyl-2-linoleoylglycerol (CLC), **C** 1,2-dilinoleoyl-3-capryloylglycerol (LLC), and **D** 1,3-dilinoleoyl-2-capryloylglycerol (LCL)

To confirm the successful synthesis and fatty acid composition of the MLCTs, molecular weights were verified using HPLC–ESI–MS analysis. As shown in Fig. 4, the mass-to-charge (*m*/*z*) of the spectrum corresponds to each MLCT. CCL was detected at 19.49 min with a *m*/*z* value of 624.6, corresponding to the expected molecular weight (606.9 g/mol) with an ammonium adduct ([M + NH₄]⁺). Similarly, CLC was detected at 19.50 min with an *m*/*z* value of 624.5, also matching the calculated mass. LLC and LCL were both detected at 21.54 min, and their *m*/*z* values were observed at 760.6, consistent with the expected molecular weight of 743.1 g/mol ([M + NH₄]⁺).

#### NMR analysis

**Fig. 5:**
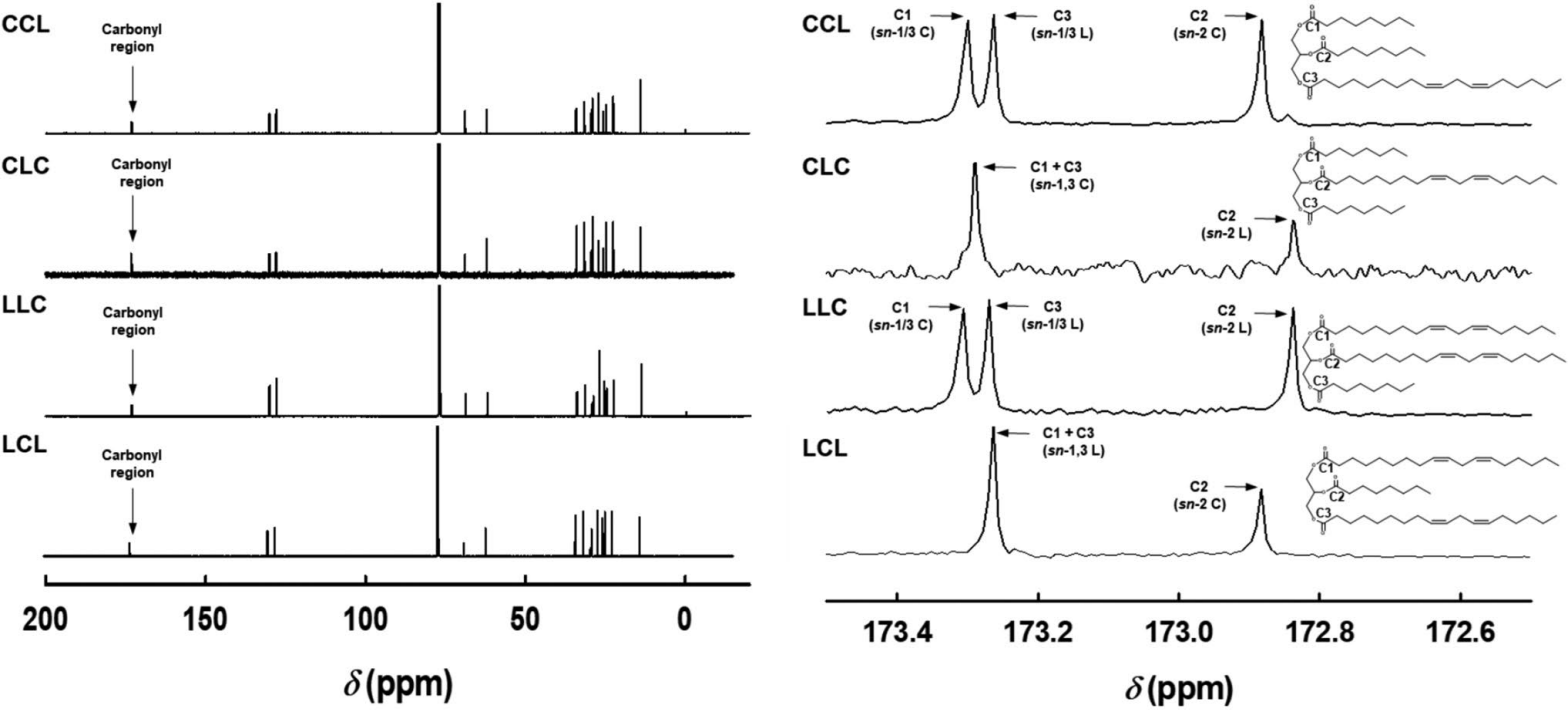
^13^C NMR spectra and carbonyl region signals of medium- and long-chain triacylglycerols. *CCL* 1,2-dicapryloyl-3-linoleoylglycerol, *CLC* 1,3-dicapryloyl-2-linoleoylglycerol, *LLC* 1,2-dilinoleoyl-3-capryloylglycerol, *LCL* 1,3-dilinoleoyl-2-capryloylglycerol. CCL (*δ*/ppm): 173.30 (1C, O*C*=O in *sn*-1/3 **C**), 173.26 (1C, O*C*=O in *sn*-1/3 **L**), 172.88 (1C, O*C*=O in *sn*-2 **C**), 130.22–127.88 (4C, =*C*H in **L**), 68.85 (1C,CH_2_*C*HCH_2_), 62.10 (1C, **L**-*C*H_2_CHCH_2_), 62.09 (1C, CH_2_CH*C*H_2_-**C**), 34.20 (1C, *C*H_2_COO in *sn*-2 **C**), 34.04 (1C, *C*H_2_COO in *sn*-1/3 **C**), 34.02 (1C, *C*H_2_COO in *sn*-1/3 **L**), 31.64 (2C, *C*H_2_CH_2_CH_3_ in **C**), 31.51 (1C, *C*H_2_CH_2_CH_3_ in **L**), 29.69–28.90 (8C, *C*H_2_), 27.18–25.35 (3C, *C*H_2_CH= in **L**), 24.88 (1C, *C*H_2_CH_2_COO in *sn*-2 **C**), 24.84 (1C, *C*H_2_CH_2_COO in *sn*-1/3 **C**), 24.82 (1C, *C*H_2_CH_2_COO in *sn*-1/3 **L**), 22.58 (2C, *C*H_2_CH_3_ in **C**), 22.56 (1C, *C*H_2_CH_3_ in **L**), 14.06–14.05 (3C, *C*H_3_). CLC (*δ*/ppm): 173.29 (2C, O*C*=O in *sn*-1,3 **C**), 172.84 (1C, O*C*=O in *sn*-2 **L**), 130.22–127.86 (4C, =*C*H in **L**), 68.86 (1C,CH_2_*C*HCH_2_), 62.07 (2C, *C*H_2_CH*C*H_2_ in *sn*-1,3 **C**), 34.18 (1C, *C*H_2_COO in *sn*-2 L), 34.03 (2C, *C*H_2_COO in *sn*-1,3 **C**), 31.63 (2C, *C*H_2_CH_2_CH3 in **C**), 31.51 (1C, *C*H_2_CH_2_CH_3_ in **L**), 29.68–28.89 (8C, *C*H_2_), 27.18–25.61 (3C, *C*H_2_CH= in **L**), 24.86 (1C, *C*H_2_CH_2_COO in *sn*-2 **L**), 24.84 (2C, *C*H_2_CH_2_COO in *sn*-1,3 **C**), 22.58 (1C, *C*H_2_CH_3_ in **C**), 22.56 (2C,*C*H_2_CH_3_ in **L**), 14.04 (3C,*C*H_3_). LLC (*δ*/ppm): 173.30 (1C, O*C*=O in *sn*-1/3 **C**), 173.26 (1C, O*C*=O in *sn*-1/3 **L**), 172.84 (1C, O*C*=O in *sn*-2 **L**), 130.21–127.87 (8C, =*C*H), 68.86 (1C,CH_2_*C*HCH_2_), 62.11 (1C, 1C, **L**-*C*H_2_CHCH_2_), 62.09 (1C, CH_2_CH*C*H_2_-**C**), 34.18 (1C, *C*H_2_COO in *sn*-2 **L**), 34.04 (1C, *C*H_2_COO in *sn*-1/3 **C**), 34.02 (1C, *C*H_2_COO in *sn*-1/3 **L**), 31.63 (2C, *C*H_2_CH_2_CH_3_ in **C**), 31.50 (1C, *C*H_2_CH_2_CH_3_ in **L**), 29.69–28.90 (4C, *C*H_2_), 27.18–25.36 (6C, *C*H_2_CH= in **L**), 24.86 (1C, *C*H_2_CH_2_COO in *sn*-2 **L**), 24.84 (1C, *C*H_2_CH_2_COO in *sn*-1/3 **C**), 24.82 (1C, *C*H_2_CH_2_COO in *sn*-1/3 **L**), 22.58 (1C, *C*H_2_CH_3_ in **C**), 22.55 (2C, *C*H_2_CH_3_ in **L**), 14.06–14.05 (3C, *C*H_3_). LCL (*δ*/ppm): 173.26 (2C, O*C*=O in *sn*-1,3 **L**), 172.88 (1C, O*C*=O in *sn*-2 **C**), 130.22–127.86 (4C, =*C*H), 68.85 (1C,CH_2_*C*HCH_2_), 62.10 (2C, *C*H_2_CH*C*H_2_ in *sn*-1/3 **L**), 34.20 (1C, *C*H_2_COO in *sn*-2 **C**), 34.02 (2C, *C*H_2_COO in *sn*-1/3 **L**), 31.63 (2C, *C*H_2_CH_2_CH3 in **L**), 31.50 (1C, *C*H_2_CH_2_CH_3_ in **C**), 29.69–28.89 (4C, *C*H_2_), 27.18–25.35 (6C, *C*H_2_CH= in **L**), 24.88 (1C, *C*H_2_CH_2_COO in *sn*-2 **C**), 24.82 (2C, *C*H_2_CH_2_COO in *sn*-1,3 **L**), 22.58 (1C, *C*H_2_CH_3_ in **C**), 22.56 (2C,*C*H_2_CH_3_ in **L**), 14.05–14.04 (3C,*C*H_3_)

To confirm that each synthesized MLCT corresponded to its intended regioisomeric structure, ^13^C NMR spectroscopy was employed. The chemical shifts observed for each compound are presented in Fig. 5. Regioisomer discrimination was facilitated by various structural influences, including long-range de-shielding effects from polyunsaturated alkene side chains, molecular conformation (steric effects), and molecular symmetry (positional effects), all of which induce characteristic changes in chemical shifts across multiple regions of the spectrum (Padley et al., 1986). Notably, it was reported that the de-shielding effect of the carbonyl function extended over six carbon–carbon single bonds (Chandler et al., 2001). Linoleic acid, with double bonds in proximity to the carbonyl group, exhibited stronger de-shielding than the fully saturated caprylic chains.

In symmetrical TAGs, the *sn*-1 and *sn*-3 acyl chains are magnetically equivalent, leading to identical ^13^C NMR resonances. Conversely, asymmetrical isomers exhibit magnetically distinct environments, resulting in differentiated signals. This combination of effects yielded notable differences in the number and position of signals in the carbonyl, glyceryl, and aliphatic regions of the ^13^C NMR spectra (Fauconnot et al., 2006). In our results, CCL exhibited three carbonyl signals at 173.30 (*sn*-1/3 C moiety), 173.26 (*sn*-1/3 L moiety), and 172.88 ppm (*sn*-2 C moiety), consistent with an asymmetric structure. In contrast, CLC showed two signals at 173.29 (*sn*-1,3 C moiety) and 172.84 ppm (*sn*-2 L moiety), corresponding to a symmetric TAG. Similarly, LLC gave three signals at 173.29 (*sn*-1/3 C moiety), 173.26 (*sn*-1/3 L moiety), and 172.84 ppm (*sn*-2 L moiety), while LCL showed two signals at 173.29 (*sn*-1,3 L moiety) and 172.88 ppm (*sn*-2 C moiety). Taken together with the HPLC–ESI–MS results, the NMR data confirm that all four MLCT congeners were successfully synthesized with the correct molecular structures.

Previous reports have shown that enzymatically synthesized MLCT blends exhibit appreciable oxidative stability in food systems and that infant formula containing MLCTs improves lipid absorption and growth outcomes, supporting their potential applications in specialized nutrition (Chen et al., 2025; Koh et al., 2009). In addition, certain MLCT isomers, such as CLC, may exhibit distinct digestive characteristics, leading to the investigation of the metabolic and physiological effects of each MLCT type through in vivo and clinical studies (Liu et al., 2022). Extending this synthetic strategy to incorporate other bioactive fatty acids may broaden its potential applications in functional foods, nutraceuticals, and specialized medical nutrition.

In this study, MLCTs with high nutritional value were successfully synthesized by controlling the positional distributions of fatty acids through the chemoenzymatic approach. The findings demonstrated that the synthetic pathway and positional configuration of fatty acids significantly influence the solubility and yield of MLCTs. Although the developed HPLC method cannot fully separate certain regioisomers, complementary analyses using HPLC–ESI–MS and ^13^C NMR provided accurate compositional and structural confirmation. This limitation highlights the need for further development of analytical techniques capable of simultaneously separating and quantifying all four MLCT congeners. Future research should also explore the scalability of the current process for industrial production, assess the oxidative stability and shelf-life of the MLCTs in specific food systems (e.g., infant formula).

## Authorship contributions

Jaehyeon Park: conceptualization, investigation, validation, data curation, writing—original draft, writing—review and editing. Juno Lee: writing—original draft, Writing—review and editing. Jihoon Kim: Writing—original draft, writing—review and editing. Pahn-Shick Chang: supervision, project administration, funding acquisition, conceptualization, writing—review and editing.

## Data Availability

Data will be made available on request.
